# “Hot” and “Cold” Cognition in Users of Club Drugs/Novel Psychoactive Substances

**DOI:** 10.3389/fpsyt.2021.660575

**Published:** 2021-03-24

**Authors:** George Savulich, Owen Bowden-Jones, Robert Stephenson, Annette B. Brühl, Karen D. Ersche, Trevor W. Robbins, Barbara J. Sahakian

**Affiliations:** ^1^Department of Psychiatry, School of Clinical Medicine, University of Cambridge, Cambridge, United Kingdom; ^2^Behavioural and Clinical Neuroscience Institute, University of Cambridge, Cambridge, United Kingdom; ^3^Club Drug Clinic, Central and North West London National Health Service (NHS) Foundation Trust, London, United Kingdom; ^4^University College London, London, United Kingdom; ^5^Eton College, Windsor, United Kingdom; ^6^University Hospital of Psychiatry, University of Basel, Basel, Switzerland; ^7^Department of Psychology, University of Cambridge, Cambridge, United Kingdom

**Keywords:** novel psychoactive substances, legal highs, club drugs, neuropsychology, drug addiction, emotion

## Abstract

Novel psychoactive substances (NPS) are popular “club/party” drugs that first attracted attention in the UK in 2009 and remained legal until the 2016 Psychoactive Substances Act criminalized their distribution. Unlike “traditional” illicit drugs, very little is known about the influence of their analogs on neuropsychological functioning. We characterized the cognitive and emotional profile of NPS/polydrug users using the Cambridge Neuropsychological Test Automated Battery (CANTAB) and EMOTICOM test battery in adult male (aged 20–49 years) recreational users without psychiatric comorbidities (*n* = 27; “psychonauts”), service users attending a UK specialist “Club Drug” Clinic for problematic use (*n* = 20) and healthy control volunteers without significant drug-taking histories (*n* = 35). Tasks were selected to distinguish “hot” cognitive processes that are highly influenced by emotion from “cold” cognitive processes that are largely independent of emotional influence. Both user groups reported significantly higher sensation-seeking traits compared with non-users. Recreational NPS users demonstrated more risk-taking behavior compared with controls and treatment-seeking NPS users showed poorer learning, episodic memory and response inhibition compared with the other two groups. These effects persisted, when controlling for age, intelligence, alcohol and cannabis use severity, nicotine dependence, trait anxiety, depression, childhood adversity, impulsivity, and sensation seeking. Overall, recreational NPS users showed elevated “hot” (emotion-laden) cognition in the absence of “cold” (non-emotional) cognitive deficits, whereas “cold” cognitive dysfunction was pronounced in individuals seeking treatment for problematic NPS use. High trait impulsivity and poor self-control may confer additional risk to NPS/polydrug use severity and separate those seeking treatment from those using NPS recreationally.

## Introduction

Novel psychoactive substances (NPS; formerly called “legal highs”) are compounds that are designed to mimic the effects of “traditional” illicit drugs such as amphetamines, ecstasy and cannabis (1–3). Here the term novel does not refer to newly invented substances, but rather to those that are newly synthetized (or available) (4). Other terms that have been used to describe these substances include “club drugs,” “designer drugs,” “party drugs,” “internet drugs,” and “research chemicals.” Recent drug monitoring reports have indicated an increase in the use, availability and harmful effects of NPS (5, 6), with the total number of new drugs being monitored globally reaching 892 in 2018 (7).

NPS can be broadly grouped into four main categories based on their mechanisms of action: stimulants (e.g., cathinones, piperazines, and phenethylamines), depressants (benzodiazepines, e.g., diclazepam and opioids, e.g., novel fentanyls), hallucinogens (dissociatives, e.g., methoxetamine and hallucinogens, e.g., 2C-series) and synthetic cannabinoids (e.g., CB_1_ and CB_2_ receptor agonists) (1, 8–11). While most established illicit drugs have distinct effects, many NPS are part of groups of similar substances with relatively similar effects (e.g., cathinones) or dissimilar substances with similar effects (e.g., synthetic cannabinoids) (12). The pharmacological profiles of NPS within the same classification can also resemble different established substances (e.g., cocaine/MDMA- and methamphetamine-like cathinones) (13). NPS are frequently recognized as one component of polysubstance use, which is generally added to, rather than replace, existing drug-taking repertoires (14, 15). NPS users are generally male (16–18) and from urban areas, with subgroups including adolescents and young adults (15–25 years), students and clubbers (19, 20). Gay and bisexual men are also often amongst the “early adopters” of new “club drug” trends (21). So-called “psychonauts” (sometimes called “cyber-psychonauts” or “e-psychonauts”) are an additional key group. They are well-educated and highly informed NPS users, who possess detailed pharmacological/pharmaceutical knowledge about the drugs they take (22). “Psychonauts” are known to experiment with combinations of hallucinogenic drugs, often sharing detailed records of their experiences online (23–25). Although some NPS users are driven by drug-specific effects (e.g., hallucinogens for self-exploration/spiritual attainment and stimulants for social enhancement), online surveys have identified curiosity, pleasure, and enjoyment as the main motivations for use (16, 26, 27). Ease of access, affordability and low detectability in drug screening tests are also important motivators (17, 28).

An increasing number of people receive treatment for NPS-related harms in the UK (29). These include acute toxicity, risky sexual and drug-sharing behaviors and adverse psychiatric, cardiovascular, renal and gastrointestinal effects (18, 30). Cognitive alterations have been well-documented in substance use disorders and predict treatment outcome (31). However, unlike “traditional” illicit drugs, very little is known about the influence of their novel analogs on neuropsychological functioning. We aimed to redress this by comparing cognitive and emotional functions in recreational NPS users without psychiatric comorbidities (*n* = 27; “psychonauts”), service-users attending a UK specialist “Club Drug” Clinic for problematic use (*n* = 20) and healthy control volunteers without significant drug-taking histories (*n* = 35). On the basis of previous research showing high risk-related behavior in NPS users (32), we hypothesized that our NPS groups would show elevated “hot” (emotion-laden) cognition compared with controls. We further hypothesized that our treatment-seeking NPS group would show broad “cold” (non-emotional) cognitive deficits compared with the other two groups, reflecting greater substance use severity and/or comorbid psychopathology typical of NPS/polydrug users in treatment (33, 34). Lastly, we explored reasons for NPS/club drug use, hypothesizing that consistent with previous studies (16, 26, 27), curiosity and experience seeking would be the main motivations in both user groups.

## Materials and Methods

### Participants

Treatment-seeking NPS users were referred from the NHS Central North West London (CNWL) “Club Drug” Clinic (www.clubdrugclinic.cnwl.nhs.uk). Recreational NPS users (psychonauts) were recruited from the community *via* posted flyers, online (e.g., advertisement on NPS forums), psychedelic/psychonauts societies and word-of-mouth. We were primarily interested in individuals who were positive about their lifestyle and experimented with NPS for a novel or unusual experience. Healthy control volunteers were recruited *via* mailing lists, posted flyers and from the Behavioral and Clinical Neuroscience Institute research volunteer database. All participants gave written informed consent.

Inclusion criteria were male, English-speakers, between 18 and 49 years of age. Participants in the recreational group reported NPS use at least twice a month in the past 3 months. Participants in the treatment-seeking group were currently receiving health care services for problematic NPS use as defined by ICD-10 criteria for harmful or dependent use (35). Exclusion criteria for the recreational NPS group were current psychiatric disorder; current harmful alcohol use as defined by a score ≥15 (36, 37) on the Alcohol Use Disorders Test [AUDIT; (38)]; current harmful cannabis use as defined by a score ≥13 on the Cannabis use Disorders Test-Revised [CUDIT-R; (39)]; and current psychoactive medication. Exclusion criteria for the treatment-seeking NPS group were current or past acute or drug-induced psychosis; current antipsychotic medication; and current infectious disease. Exclusion criteria for healthy controls were current or past neurological or psychiatric conditions; current or past substance use (excluding low-risk alcohol use as defined by an AUDIT score <8; <10x lifetime cannabis use was permitted, but only if not used in the past 6 months); and current psychoactive medication. Additional exclusion criteria for all participants were past serious traumatic head injury and severe physical impairments affecting eyesight or motor performance.

### Measures

#### Substance Use

Alcohol and cannabis use severity were measured using the AUDIT (38) and CUDIT-R (39). Nicotine dependence was measured using the Fagerström Test for Nicotine Dependence [FTND; (40)], which assesses the quantity of cigarette consumption, the compulsion to use and dependence. Reasons for NPS/club drug use were explored in the two user groups by asking them to endorse up to 34 fixed reasons (e.g., pleasure/enjoyment; facilitation of social situations; stress relief) using a semi-structured interview.

#### Psychopathology

Current symptoms of attention deficit/hyperactivity disorder (ADHD) were assessed with the Adult ADHD Self-Report Scale [ASRS; (41)]. Anxiety and depression were assessed with the Spielberger Trait Anxiety Inventory [STAI; (42)] and Beck Depression Inventory [BDI-II; (43)]. Childhood adversity was assessed with the Childhood Trauma Questionnaire [CTQ; (44)].

#### Impulsivity and Sensation Seeking

Impulsivity was assessed with the Barratt Impulsiveness Scale [BIS-11; (45)]. Sensation seeking, including thrill and adventure seeking (e.g., participation in highly stimulating physical activities), disinhibition (e.g., varied sexual and/or drug-seeking behaviors), experience seeking (e.g., pursuit of novel and/or unconventional experiences), and boredom susceptibility (e.g., aversion to repetition and/or prediction) were assessed with the Sensation Seeking Scale [SSS-V; (46)].

### Neuropsychological Functioning

Premorbid IQ was estimated using the National Adult Reading Test [NART; (47)]. Standardized neuropsychological tasks were selected from the reliable and well-validated EMOTICOM neuropsychological test battery (48) and Cambridge Neuropsychological Test Automated Battery (CANTAB; www.cambridgecognition.com).

#### “Hot” Cognition (Impulsivity and Risk-Taking Behavior)

The Discounting task (DT) (48) assesses temporal discounting across five levels of delay (0, 30, 90, 180, and 365 days) and probability (25, 50, 75, 90, and 100%). Participants must choose between a standard fixed amount (always £20) associated with a particular delay or probability and an alternative amount available immediately. Indifference points are calculated for each length of delay and degree of uncertainty. These refer to the amount of immediately available money that the participant considered to be equivalent to the delayed or uncertain reward. Area under the curve (AUC) was calculated using the following formula:

AUC *(for delay discounting)* = [(2-0)^*^((indifference point at 0 days + indifference point at 2 days)/2)] + [(30-2)^*^((indifference point at 2 days + indifference point at 30 days)/2) + [(180-30)^*^((indifference point at 3 days + indifference point at 180 days)/2)] + [(365−180)^*^((indifference point at 180 days+ indifference point at 365)/2)].

A smaller AUC indicates more severe discounting of the delayed reward (i.e., greater impulsivity). A similar formula was used for probability discounting, with a smaller AUC indicating greater risk aversion.

The Cambridge Gambling task (CGT) (48) assesses risk-taking behavior outside of a learning context. Participants are shown a roulette wheel with two different proportions of colors. Participants are asked to place a monetary bet on the outcome they expect (i.e., which color the wheel pointer will land on when spun). There are five different wheel proportions, ranging from very certain to very uncertain. In the win (reward) condition, participants either double (if they win) or retain (if they lose) their bet. In the loss condition, participants lose their bet if they make the wrong selection. Risk adjustment quantifies bet calibration across ratios (calculated for the reward and loss conditions separately) using the following formula (a higher score is preferable):

*Risk adjustment* = (2^*^bet at 90%) + (1^*^bet at 80%) + (0^*^bet at 70%) – (1^*^bet at 60%) – (2^*^bet at 50%)/Average bet.

#### “Cold” Cognition (Memory, Attention, and Executive Functions)

Learning and episodic memory were assessed using the Paired Associates Learning (PAL) task (49). Boxes are displayed on a screen and opened in a randomized order, some of which contain a pattern. The participant must touch the box where they think the pattern was originally located. If an error is made, then the patterns are presented again. Outcome measures include the total number of errors made, the total number of trials required to locate all of the patterns correctly and first trial memory score (i.e., the number of patterns correctly located after the first trial summed across the number of stages completed).

Attention and executive functions were measured using the Rapid Visual Information Processing (RVP) task, the Spatial Working Memory (SWM) task, and the Stop-Signal Task (SST). The RVP task assesses sustained visual attention (50). Single digits appear in the center of the screen at a rate of 100 digits per minute. Participants must detect a series of three targets (e.g., 3-5-7) by pressing a button as quickly as possible. Sensitivity detecting the target sequence regardless of response tendency (A') is the main outcome measure.

The SWM task assesses ability to retain spatial information and manipulate remembered items in working memory (51). Using a process of elimination, participants are instructed to find a yellow token in boxes appearing on the screen. The position of the boxes changes and the number of boxes increases for each trial. Outcome measures include between errors (revisiting a box in which a token has previously been found) and strategy (a predetermined sequence by beginning with a specific box and then, once a token has been found, returning to that box to start a new search sequence).

The SST measures response inhibition (52). Participants first respond to an arrow stimulus by selecting a button depending on the direction in which it points (counterbalanced and intermixed). Participants are later asked to withhold making a response if a 300 Hz, 100 ms audio tone (i.e., stop-signal) is present (25% of the trials). The task uses a staircase design to adapt to the participant's performance, allowing for a 50% success rate for inhibition. Participants were instructed to respond as quickly and accurately as possible. The task includes 16 practice trials, 240 “Go” trials and 80 “stop” trials. Outcome measures include direction errors, proportion of successful stops, reaction time on “Go” trials and stop-signal reaction time (SSRT, i.e., the time taken to abort an initiated action in the presence of a stop-signal). SSRT was only estimated for participants successfully inhibiting 25–75% of their responses (53).

### Procedure

This study received University of Cambridge Psychology research ethics approval (Pre.2015.51; Pre.2020.087) and East of England Cambridge Central research ethics committee approval (ref 17/EE/0453). Volunteers in the recreational NPS and healthy control groups were screened for study criteria by telephone and *via* semi-structured interview using the Mini-International Neuropsychiatric Inventory [MINI; (54)]. Volunteers in the treatment-seeking NPS user group were referred by keyworkers from the CNWL Club Drug Clinic. Participants of each group were recruited in parallel. Invited participants attended a single 3-h session at the Behavioral and Clinical Neuroscience Institute, their home or the clinic. Participants were asked to abstain from alcohol consumption 24 h prior to the experiment and nicotine from the morning of the session. On arrival participants completed basic demographic and substance use measures. Neuropsychological tests were then administered in a fixed order. Participants then completed questionnaire measures, followed by a semi-structured interview to assess reasons for drug/club drug use. Volunteers were offered breaks as necessary (smoking was permitted after tests of neuropsychological functioning) and paid for their participation.

### Statistical Analyses

Analyses were conducted using SPSS 27.0. Demographic, substance use and trait measures were analyzed by group using one-way analysis of variance (ANOVA) or chi-square tests as appropriate. Three-group comparisons were made for each neuropsychological test measure using univariate analysis of covariance (ANCOVA), covarying for age, intelligence (IQ; NART), alcohol and cannabis use severity (AUDIT, CUDIT-R), nicotine dependence (FTND), trait anxiety (STAI), depression (BDI-II), childhood adversity (CTQ), impulsivity (BIS-11), and sensation seeking (SSS-V). These measures were selected to better isolate any observed effects to the influence of NPS/comorbid polydrug use. The Benjamini-Hochberg procedure was applied at *q* < 0.15 to control for false discovery for each neuropsychological test battery; all significant *p*-values remained (two-sided). For models reaching significance, planned pairwise comparisons of covariate-adjusted means were followed-up *post-hoc*. Correlational analyses between “hot” cognitive processes and sensation seeking and impulsivity were explored.

## Results

### Demographic, Psychopathology, Substance Use, and Personality Trait Measures

The three groups were well-matched in age, IQ and years in education. However, fewer of those seeking treatment were currently employed or studying (Table 1). Participants in the treatment-seeking NPS group met ICD-10 criteria (35) for mental and behavioral disorder due to harmful cocaine (5%), cannabis (10%), and hallucinogen use (5%); mental and behavioral disorder due to dependent sedative/hypnotic (15%), volatile solvent (10%), and stimulant (35%) use. Harmful ketamine (20%), alcohol (5%), cocaine (5%), benzodiazepine (5%), and other psychoactive substance (5%) use were also reported. Psychiatric diagnoses in the treatment-seeking NPS group were generalized anxiety disorder (30%), depression (30%), borderline personality disorder (10%), and panic disorder (5%). Current prescribed medications were mirtazapine (15%), propranolol (10%), diazepam (10%), fluoxetine (5%), sertraline (5%), citalopram (5%), alprazolam (5%), codeine (5%), and naltrexone (5%). Medications that were taken, but not prescribed, were diazepam (20%) and propranolol (5%).

**Table 1:** Demographic information, psychopathology, trait measures, substance use severity (means and standard deviations), and percentages of novel psychoactive and comorbid polysubstance use in the past 90 days by group.

	**Control group *n* = 35**	**Recreational group *n* = 27**	**Treatment-seeking group *n* = 20**	**Statistic, *p-*value, effect size**
**Demographics**				
Age (years)	26.43 (6.84)	25.11 (3.90)	28.95 (5.11)	*F*_(2,79)_ = 2.73, *p* = 0.07, *η^2^* = 0.07
IQ (NART)	116.17 (6.20)	117.00 (6.79)	114.10 (7.55)	*F*_(2,79)_ = 1.10, *p* = 0.34, *η^2^* = 0.03
Education (years)	15.20 (2.18)	14.78 (2.03)	14.15 (1.81)	*F*_(2,79)_ = 1.68, *p* = 0.19, *η^2^* = 0.04
In work or education (%)	100%	96%	40%	***X*****^2^ = 38.80**, ***p*** ** < 0.001, V = 0.69**
**Psychopathology**				
Trait Anxiety (STAI-T)	38.86 (10.78)	39.32 (10.69)	54.00 (11.11)	***F***_**(2, 77)**_ **= 14.29**, ***p*** ** < 0.001**, ***η^2^*** **= 0.27**
Depression (BDI-II)	6.69 (7.67)	9.20 (8.65)	22.37 (13.35)	***F***_**(2, 76)**_ **= 17.27**, ***p*** ** < 0.001**, ***η^2^*** **= 0.31**
ADHD symptoms (ARSC)	1.97 (1.60)	1.92 (1.61)	4.05 (1.39)	***F***_**(2, 76)**_ **= 13.20**, ***p*** ** < 0.001**, ***η^2^*** **= 0.26**
Childhood adversity (CTQ)	43.91 (10.83)	48.76 (15.58)	63.74 (6.67)	***F***_**(2, 76)**_ **= 17.59**, ***p*** ** < 0.001**, ***η^2^*** **= 0.32**
**Impulsiveness/sensation seeking**				
Impulsivity (BIS-11)	60.11 (11.15)	64.52 (9.39)	73.15 (8.04)	***F***_**(2, 77)**_ **= 11.01**, ***p*** ** < 0.001**, ***η^2^*** **= 0.22**
Sensation seeking (SSS-V total)	19.56 (6.95)	26.72 (4.67)	25.58 (5.03)	***F***_**(2, 75)**_ **= 12.65**, ***p*** ** < 0.001**, ***η^2^*** **= 0.25**
Thrill/adventure seeking	6.24 (2.19)	6.88 (2.71)	6.63 (2.52)	*F*_(2,75)_ = 0.41, *p* = 0.67, *η^2^* = 0.01
Disinhibition	4.26 (2.57)	7.04 (1.67)	6.95 (2.04)	***F***_**(2, 75)**_ **= 14.94**, ***p*** ** < 0.001**, ***η^2^*** **= 0.29**
Experience seeking	6.03 (1.75)	8.24 (1.69)	7.79 (1.58)	***F***_**(2, 75)**_ **= 14.00**, ***p*** ** < 0.001**, ***η^2^*** **= 0.27**
Boredom susceptibility	3.03 (1.75)	4.56 (1.66)	4.21 (1.81)	***F***_**(2, 75)**_ **= 12.65**, ***p*** ** < 0.001**, ***η^2^*** **= 0.14**
**Substance use severity**				
Age of first NPS use (years)		20.33 (3.22)	21.00 (5.86)	*t*_(45)_ = 0.50, *p* = 0.62, *η^2^* = 0.01
Alcohol use (AUDIT)	3.91 (2.44)	5.70 (3.45)	11.40 (9.41)	***F***_**(2, 79)**_ **= 13.13**, ***p*** ** < 0.001**, ***η^2^*** **= 0.25**
Cannabis use (CUDIT-R)	0.00 (0.00)	4.19 (3.54)	8.20 (7.34)	***F***_**(2, 79)**_ **= 25.82**, ***p*** ** < 0.001**, ***η^2^*** **= 0.40**
Nicotine use (FTND)	0.37 (1.01)	0.37 (1.26)	1.60 (2.19)	***F***_**(2, 79)**_ **= 5.27**, ***p*** **= 0.01**, ***η^2^*** **= 0.19**
Drug-sharing behavior (%)		19%	53%	***X*****^2^ = 5.91**, ***p*** **= 0.02, V = 0.36**
Drug-injecting behavior (%)		11%	37%	***X*****^2^ = 4.34**, ***p*** **= 0.04, V = 0.31**
**Novel psychoactive substance use (% past 90 days)**				
Ketamine		5.7%	60%	
Nitrous oxide		44.4%	10%	
Mephedrone		7.4%	30%	
2C (2CB, 2CE)		29.6%	10%	
1-propionyl-lysergic acid diethylamide		25.9%	0%	
Synthetic cannabinoids		3.7%	20%	
Gamma Hydroxybutyrate		0%	15%	
Methoxetamine		0%	15%	
Alkyl nitrites (poppers)		14.8%	0%	
Ethylphenidate		0%	10%	
*Salvia divinorum*		0%	10%	
25I-NBOMe		0%	10%	
Kratom (*Mitragyna specios*)		7.4%	0%	
Methylone		0%	5%	
Kratom		0%	5%	
Other psychedelics not specified		0%	5%	
Methoxydine		3.7%	0%	
Dimethyltryptamine		3.7%	0%	
Kava		3.7%	0%	
Kanna		3.7%	0%	
**Comorbid polysubstance use (% past 90 days)**				
Alcohol		100%	85%	
Cocaine		18.5%	80%	
Cannabis		66.6%	55%	
Diazepam		7.4%	65%	
Ecstasy		33.3%	35%	
Amphetamine		14.8%	40%	
Methamphetamine		0%	10%	
Crack cocaine		0%	10%	
Heroin		0%	10%	
Lysergic acid diethylamide		3.7%	5%	

*NART, National Adult Reading Test; AUDIT, Alcohol Use Disorders Identification Test; CUDIT-R, Cannabis Use Disorder Identification Test-Revised; FTND, Fagerström Test of Nicotine Dependence; BDI-II, Beck Depression Inventory; STAI, Spielberger Trait Anxiety Inventory; ARSC, Adult ADHD Self-Report Scale; CTQ, Childhood Trauma Questionnaire, BIS-11, Barratt Impulsiveness Scale; SSS-V, Sensation Seeking Scale. Bold indicates p < 0.05*.

All participants in the NPS groups reported using combinations of novel psychoactive and illicit substances (Table 1). Age of first NPS use did not significantly differ between the two user groups (*p* = 0.62). Self-reported severity of alcohol, cannabis and nicotine use were significantly higher in the treatment-seeking NPS group compared with the other two groups (all *p*'s < 0.007). Cannabis (*p* < 0.001), but not alcohol (*p* = 0.19) or nicotine (*p* = 0.10) use severity significantly differed between the recreational NPS and control groups. Nicotine use was generally low in all three groups. Drug-sharing and injecting behaviors were significantly higher in the treatment-seeking NPS group (*p*'s ≤ 0.04).

As expected, the three groups significantly differed in trait anxiety, depression, ADHD symptoms, childhood adversity, impulsivity, and sensation-seeking traits (Table 1). *Post-hoc* comparisons showed that the treatment-seeking NPS group scored significantly higher on all trait measures compared with both the recreational NPS and control groups (all *p*'s < 0.006), except for sensation seeking (total score), which did not significantly differ from the recreational NPS group [mean difference = −1.14, *p* = 0.52, 95%CI (−4.69, 2.41)]. Sensation seeking was the only trait measure that was significantly higher in the recreational NPS group compared with the control group [mean difference = 7.16, *p* < 0.001, 95%CI (4.09, 10.23)]. On sensation seeking subscales, the two NPS groups did not significantly differ (all *p*'s > 0.37). However, the NPS groups showed significantly more disinhibition, experience seeking and boredom compared with the control group (all *p*'s ≤ 0.02).

### “Hot” Cognition

**Figure 1:**
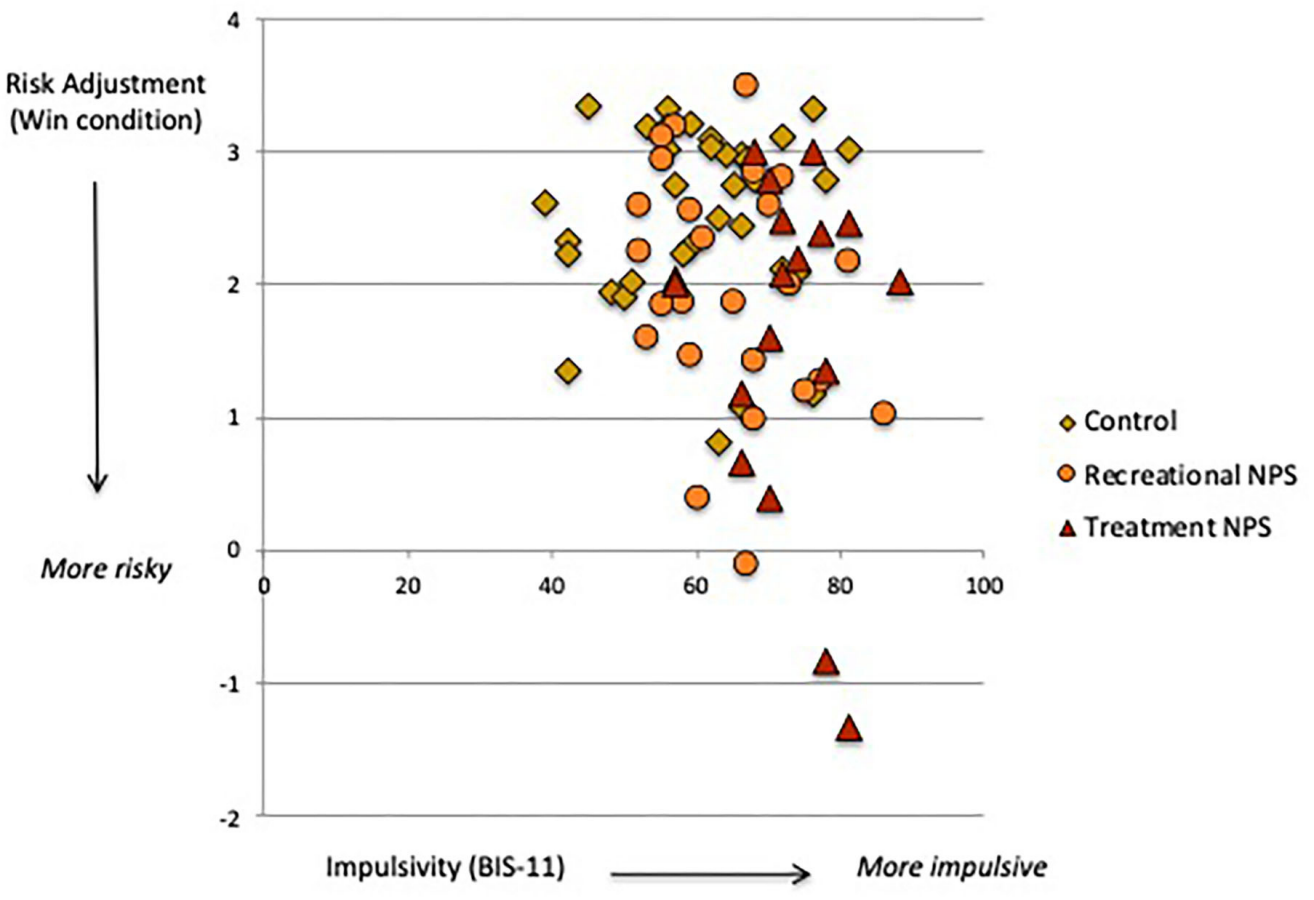
Association between trait impulsivity (BIS-11) and risk adjustment (Cambridge Gamble task, win condition).

Delay and probability discounting (DT) did not significantly differ between the three groups (Table 2). However, the recreational NPS group showed significantly worse risk adjustment (i.e., more risk-taking behavior; CGT) compared with the control group [mean difference = −0.84, *p* = 0.01, 95%CI (−1.47, −0.21)]. Risk adjustment in the recreational NPS group did not significantly differ from the treatment seeking-NPS group [mean difference = −0.25, *p* = 0.52, 95%CI (−1.02, 0.52)]. Riskier behavior by the recreational NPS group compared with controls was observed in the loss condition only. Across the entire sample, higher impulsivity was associated with riskier behavior in the win condition, *r* = −0.25, *p* = 0.03 (Figure 1). Sensation seeking (total score) was not significantly associated with riskier behavior, *r* = −0.17, *p* = 0.15.

**Table 2:** Measures of “hot” and “cold” cognitive processes (means and standard deviations) by group.

	**Control group** ***n* = 35**	**Recreational group** ***n* = 27**	**Treatment-seeking group** ***n* = 20**	**Statistic, *p*-value, effect size**
“**Hot**” **cognition**				
**Impulsivity**				
Discounting task				
Delay (area under the curve)	4631.54 (2181.80)	4641.78 (2010.98)	3738.42 (1805.34)	*F*_(2,64)_ = 1.13, *p* = 0.33, η^2^ = 0.03
Probability (area under the curve)	934.41 (317.68)	852.00 (275.44)	1035.56 (351.36)	*F*_(2,64)_ = 2.53, *p* = 0.09, η^2^ = 0.07
**Risk-taking**				
New Cambridge Gamble Task				
Risk adjustment (reward)	2.54 (0.68)	2.00 (0.90)	1.79 (0.98)	*F*_(2,64)_ = 2.05, *p* = 0.14, η^2^ = 0.06
Risk adjustment (loss)	2.54 (0.76)	1.87 (1.08)	2.14 (0.80)	***F***_**(2, 64)**_ **= 3.59**, ***p*** **= 0.03**, **η^2^ = 0.10**
“**Cold**” **cognition**				
**Memory**				
Paired Associates Learning				
Total errors	12.88 (8.89)	23.08 (19.46)	64.58 (61.57)	***F***_**(2, 65)**_ **= 7.46**, ***p*** **= 0.001**, **η^2^ = 0.19**
Total trials	9.12 (2.28)	12.20 (5.23)	17.37 (6.10)	***F***_**(2, 65)**_ **= 4.31**, ***p*** **= 0.02**, **η^2^ = 0.12**
First trial memory score	28.85 (6.31)	26.28 (7.71)	19.00 (6.91)	*F*_(2,65)_ = 2.75, *p* = 0.07, η^2^ = 0.08
**Attention**				
Rapid Visual Information Processing				
A”	0.95 (0.04)	0.92 (0.05)	0.89 (0.7)	*F*_(2,61)_ = 1.69, *p* = 0.19, η^2^ = 0.05
**Executive functions**				
Spatial Working Memory				
Between errors	14.21 (12.08)	22.96 (13.45)	32.11 (24.13)	*F*_(2,64)_ = 1.78, *p* = 0.18, η^2^ = 0.05
Strategy	18.79 (5.11)	20.21 (6.07)	23.84 (7.23)	*F*_(2,64)_ = 0.10, *p* = 0.90, η^2^ = 0.003
Stop Signal Task				
Direction errors	1.64 (2.22)	2.29 (3.54)	1.76 (1.99)	*F*_(2,61)_ = 1.39, *p* = 0.26, η^2^ = 0.04
Proportion of successful stops	0.49 (0.10)	0.48 (0.10)	0.52 (0.07)	*F*_(2,61)_ = 0.73, *p* = 0.49, η^2^ = 0.02
Reaction time on Go trials (median)	461.03 (174.39)	476.33 (145.35)	517.38 (141.66)	*F*_(2,61)_ = 0.23, *p* = 0.77, η^2^ = 0.01
Stop-signal reaction time (SSRT)	163.65 (39.94)	162.91 (32.50)	194.59 (51.66)	***F***_**(2, 61)**_ **= 4.32**, ***p*** **= 0.02**, **η^2^ = 0.12**

*Analyses are controlling for age, IQ (NART), alcohol and cannabis use severity (AUDIT, CUDIT), nicotine dependence (FTND), trait anxiety (STAI-Trait), depression (BDI-II), childhood adversity (CTQ), impulsivity (BIS-11), and sensation seeking (SSS-V). Bold indicates p < 0.05*.

### “Cold” Cognition

Memory performance significantly differed between the three groups (Table 2). The treatment-seeking NPS group showed significantly poorer learning and episodic memory compared with the other two groups [PAL errors: treatment vs. recreational mean difference = 49.84, *p* = 0.001, 95%CI (20.98, 78.70); treatment vs. control mean difference = 66.22, *p* < 0.001, 95%CI (30.61, 101.83); PAL trials: treatment vs. recreational mean difference = 4.18, *p* = 0.03, 95%CI (0.46, 7.91); treatment vs. control mean difference = 6.75, *p* = 0.01, 95%CI (2.16, 11.35)]. Memory performance did not significantly differ between the recreational NPS and control groups [errors: mean difference = 16.38, *p* = 0.18, 95%CI (−7.93, 40.70); trials: mean difference = 2.57, *p* = 0.11, 95%CI (−0.57, 5.71)].

Stop-signal reaction time (SSRT) also significantly differed between the three groups (Table 2). The treatment-seeking NPS group showed a significantly longer stopping response (i.e., poorer self-control) compared with the other two groups [treatment vs. recreational mean difference = 34.38, *p* = 0.04, 95%CI (1.78, 66.97); treatment vs. control mean difference = 59.33, *p* = 0.01, 95%CI (18.94, 99.73)]. SSRT did not significantly differ between the recreational NPS and control groups [mean difference = 24.96, *p* = 0.08, 95%CI (−2.66, 52.58)]. Three-group comparisons for measures of attention (RVP) and spatial working memory (SWM) were not significant (*p*'s > 0.17) and therefore not followed-up.

### Reasons for NPS/Club Drug Use

**Figure 2:**
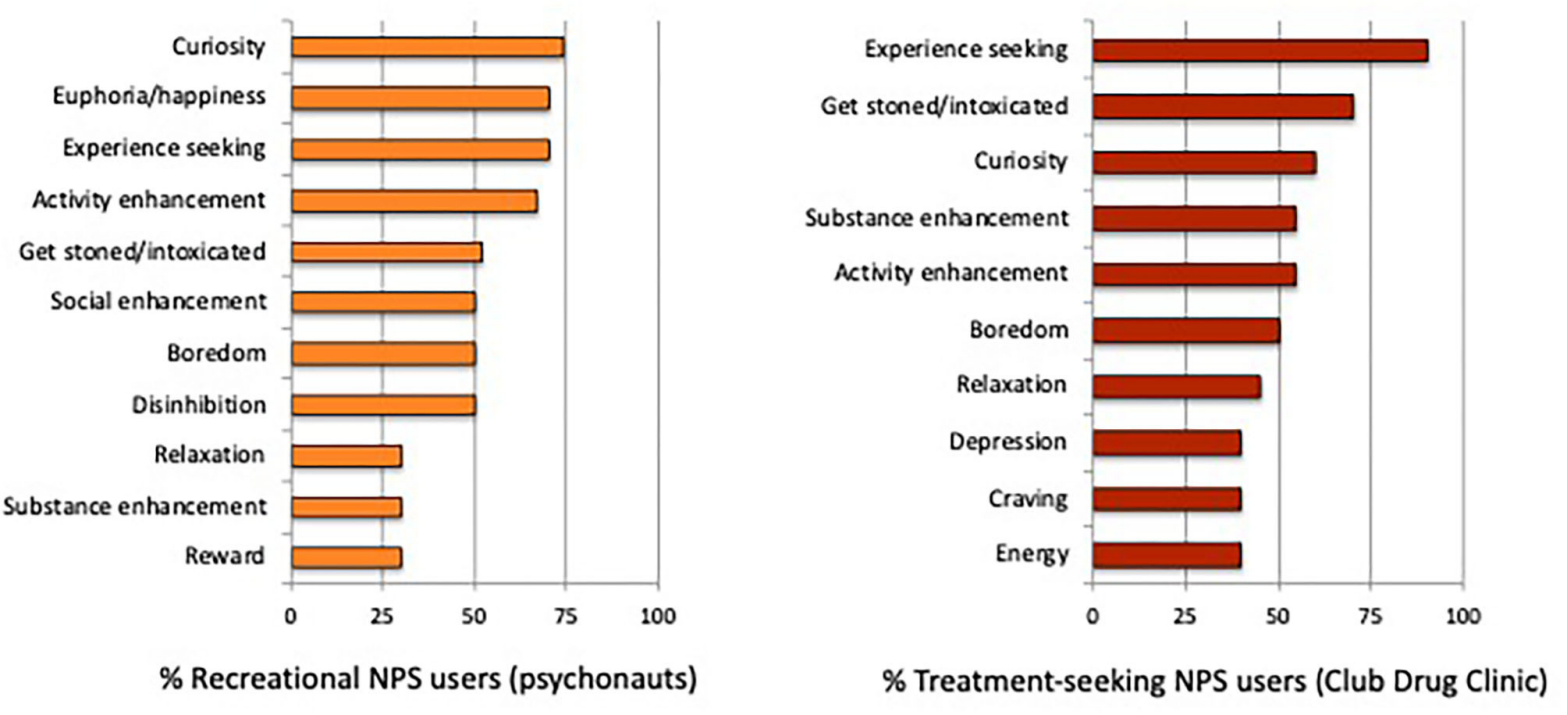
Endorsed reasons for NPS/club drug use by (%) group.

In the recreational NPS group, the top three reasons for use were (1) “*out of curiosity to see what effects they might have on me”* (74%); and (2 and 3 [tied]) “*to feel elated or euphoric”* and “*to get an unusual experience, or experience a different state of mind”* (70%). In the treatment-seeking NPS group, the top three reasons for use were: (1) “*to get an unusual experience, or experience a different state of mind”* (90%); (2) “*to get really stoned or intoxicated”* (70%); and (3) “*out of curiosity to see what effects they might have on me”* (60%). Additional reasons for NPS/club drug use are presented in Figure 2.

## Discussion

We conducted, to our knowledge, the first quantitative study investigating the relationship between NPS/polydrug use and neuropsychological functioning. Carefully controlling for an array of key demographic, substance-related and trait measures likely to impact neuropsychological functioning, we firstly found that recreational NPS users showed significantly worse risk adjustment compared with controls. We secondly found that treatment-seeking NPS users showed significantly poorer learning, episodic memory, and response inhibition compared with the other two groups. Together, these findings indicate that risk-taking behavior was higher in recreational NPS users, whereas group differences in episodic memory and inhibitory control processes were driven by those seeking treatment, suggesting possible dissociable effects of NPS/polydrug use severity on “hot” and “cold” cognitive processes.

### Elevated Risk-Taking Behavior in Psychonauts

Recreational NPS users showed riskier behavior on our gambling task (CGT), specifically toward a potential negative outcome. NPS use is associated with risky behaviors (30), which may reflect alterations in “hot” decision-making related to searching for excitement, taking risks for the sake of new experiences and/or lower perception of drug-related risks (32, 55). More risk taking in the loss condition suggests that recreational NPS users may be less sensitive to negative outcomes or more optimistic about their decision-making. They may also exhibit “loss-chasing” behavior whereby betting becomes more amplified in an effort to recover prior losses (56). It is important to note that sensation seeking and impulsivity are two personality traits that are prominent in substance-dependent individuals (55, 57). Whereas, both of our NPS user groups reported high sensation seeking, those seeking treatment were additionally characterized by high trait impulsivity. We further found that across our sample, higher levels of trait impulsivity (and *not* sensation seeking) were associated with more risk taking toward potential reward (see Figure 1). The lack of correlation with sensation seeking is likely due to its dimensions not explicitly relating to non-social rewards, including monetary earnings used in this task. The significant association between trait impulsivity and risk-taking in the win condition indicates an approach response for rewarding stimuli. This likely reflects rash impulsiveness, a dimension of impulsivity involving difficulty inhibiting one's behavior when engaging in rewarding activities (58). Rash impulsiveness is thus a key personality trait that promotes risk-taking behavior through activation of unplanned and immediate decision-making.

### “Cold” Cognitive Deficits in Treatment-Seeking NPS Users

In contrast to elevated “hot” cognition, we did not find impaired “cold” cognition in our recreational NPS group relative to non-users, which likely reflects their overall good general health. Indeed, the recreational NPS users in our sample reported mainly using novel hallucinogens, the class of NPS associated with the lowest harm profile (1). “Psychonauts” are well-educated and knowledgeable about the NPS they use, with an almost academic-like interest in thinking about hallucinogenic drug experiences (59, 60). This aligns with highly intelligent and socially integrated recreational users who incorporate regular drug taking into their lives without transitioning to dependence (61), suggesting shared resilience factors (62) such as demographic (e.g., intelligence) and/or personality features (e.g., low impulsiveness). Our data provide the first behavioral evidence that recreational NPS users demonstrate higher risk-taking in the absence of core “cold” cognitive deficits, which may protect against more adverse consequences of chronic NPS/polydrug use.

A more severe profile of “cold” cognitive dysfunction was observed in our treatment-seeking NPS group compared with both the recreational NPS and control groups. Specifically, they made more errors and needed more trials to learn the correct associations between a stimulus and a spatial location on our memory task (PAL). Impaired episodic memory is highly prevalent in male substance users (63). Difficulties forming paired associations strongly relate to structural changes in the hippocampal formation, a key region that is amongst the first to be altered by the course of drug addiction (64). Our treatment-seeking NPS group further showed difficulty with self-control. Inhibitory control is a core executive function subserved by cortical and subcortical structures including the inferior frontal gyrus (65). Similar to previous studies in stimulant drug users, we found a slower stopping response (SSRT) in the presence of intact psychomotor speed (“Go” reaction time) (66) in the treatment-seeking NPS users. Stopping response in the recreational NPS users was comparable with controls, suggesting good self-control in both groups. Of strong clinical interest, the significant difference in SSRT performance between the NPS user groups suggests that the *inability to stop* a prepotent response once initiated separated those seeking treatment from those using NPS recreationally. Impulsivity and lack of self-control (SSRT) are vulnerability factors that may predate drug taking, rendering some individuals more susceptible to developing problematic use (67). This is in stark contrast with sensation-seeking traits, which may contribute to the initiation of NPS use, but not escalation to dependence.

### Reasons for NPS/Club Drug Use

Reasons for NPS/club drug use corresponded with those reported by previous studies (16, 26, 27), and mainly reflected curiosity, experience seeking and the desire to get high and/or intoxicated. However, some distinct motivational patterns of NPS use emerged by group. Similar to hallucinogen drug users (68), inducing feelings of euphoria (i.e., pleasure/happiness) was specific to recreational NPS users. In those seeking treatment, feeling depressed/down and drug craving were reported, indicating some degree of negatively reinforced NPS use. Coping with negative emotions is one motivational pathway that could be targeted by treatment interventions for problematic NPS use. Substance enhancement (i.e., intensifying combined effects) was also more prevalent in our treatment-seeking NPS group, which can facilitate increased polydrug use associated with negative emotional states.

It is worth noting that as expected, psychopathology was high in our treatment-seeking NPS group, with over half the sample (55%) meeting criteria for at least one psychiatric diagnosis. Case studies have reported that NPS use can induce psychiatric symptoms in individuals with no prior mental health difficulties and exacerbate symptoms in those with severe mental illnesses (33). Although we were unable to determine if psychiatric symptoms predate or emerge as a consequence of NPS use (or some other contributing factor, such as childhood adversity), the high prevalence of comorbid psychopathology is consistent with more clinical features being present in individuals with higher NPS/polydrug use severity (34).

### Clinical Implications

Our study has clinical implications. Most NPS users are new to drug treatment services (19, 69), where healthcare professionals report feeling less confident managing cases of novel compared with established substances (70). As such, “club drug” clinics require specialist staff and training to better understand the complexity of NPS types, the context in which they are used and the most appropriate treatment strategies. We suggest that problematic NPS use could be screened through new tools that take into account the pattern of neuropsychological deficits observed here. For example, a “clinician-friendly” screening tool could be developed to detect impaired inhibitory control (or a more general “cold” cognitive assessment) in treatment-seeking NPS users. Self-control abilities could then be strengthened by appropriately matched interventions. In recreational NPS users, we suggest that risky behaviors (identified by “hot” cognitive assessment) could be minimized or more safely redirected as key preventative measures. Rash impulsivity and self-control could also be profiled in young adults who are at risk of harmful substance use, particularly if other vulnerability factors (e.g., social/environmental) are present.

### Limitations

The main limitation of our study is the nominal grouping of NPS users irrespective of drug class. We acknowledge that NPS classes may differentially affect physical and psychological outcomes and that our findings do not capture potential variation in cognitive performance between subgroups. Larger studies could also examine differences in cognitive performance in relation to diagnostic criteria in treatment-seeking samples (e.g., harmful vs. dependent use). Another limitation is that we did not control for treatment effects in our clinic-attending group, although treatments (e.g., antidepressant medication, cognitive behavior therapy for substance use disorders) are more likely to improve, rather than impair, cognitive functions. We also did not evaluate recency of drug use, which is likely to impact cognitive performance. Determining what makes recreational NPS users transition to problematic or dependent use compared with those who continue using recreationally is also of critical importance. Our data suggests that sensation seeking traits (predicting the initiation of NPS use) alongside high trait impulsivity and poor self-control (predicting the development of compulsive use) may be a particularly potent combination for differentiating these groups. Finally, although novel substance use is more prevalent in men (12, 18), emerging sex/gender differences in the subjective, clinical and pharmocokinetic responses to NPS pose an important avenue of new research (71).

## Conclusions

Overall, we characterized neuropsychological functioning in NPS/polydrug users, in which two distinct profiles emerged: elevated “hot” cognition (risk-taking behavior) in recreational NPS users, and impaired “cold” cognition (episodic memory, response inhibition) in treatment-seeking NPS users. Recreational NPS users without significant health and social problems may seek new or unusual experiences in the absence of “cold” cognitive deficits. They may also benefit from protective factors such as better education, more employment and good control of themselves and their drug use. In contrast, difficulties with impulsivity and self-control may confer additional risk to NPS/polydrug use severity in those seeking treatment. As treatments for substance dependence rely on sufficient neuropsychological functioning, earlier, targeted approaches are more likely to be successful in NPS users with greater use severity, before “rescuing” cognition is the only option. Synergy between schools/Universities, club/festival venues and treatment services (i.e., drug, mental, and sexual health) will be thus be crucial for identifying commonalities in harmful NPS/polydrug use and dependency. Recognizing neuropsychological deficits as another component of NPS use may also help guide preventative and harm reduction strategies, particularly for higher-risk groups.

## Data Availability Statement

The raw data supporting the conclusions of this article will be made available by the authors upon reasonable request.

## Ethics Statement

The studies involving human participants were reviewed and approved by the University of Cambridge Psychology research ethics committee (Pre.2015.51; Pre.2020.087) and East of England Cambridge Central research ethics committee (ref 17/EE/0453). The patients/participants provided their written informed consent to participate in this study.

## Author Contributions

GS collected data, analyzed data, interpreted data, and wrote the manuscript. OB-J referred subjects, interpreted data, and provided feedback on the manuscript. RS formulated the research questions, designed the study, interpreted data, and provided feedback on the manuscript. AB collected data, interpreted data, and provided feedback on manuscript. KE provided materials, interpreted data, and provided feedback on manuscript. TR formulated the research questions, designed the study, provided materials, interpreted data, and provided feedback on manuscript. BS formulated the research questions, designed the study, provided materials, interpreted data, provided feedback on the manuscript, and supervised the study. All authors contributed to the article and approved the submitted version.

## Conflict of Interest

BS consults for Cambridge Cognition, Greenfield BioVentures, and Cassava Sciences. TR consults for Cambridge Cognition, Greenfield Bioventures, Cassava Sciences, Takeda, Arcadia, Merck Sharpe and Dohme, and receives research grants from Shionogi and GlaxoSmithKline. The remaining authors declare that the research was conducted in the absence of any commercial or financial relationships that could be construed as a potential conflict of interest.
